# ARVs: The Next Generation. Going Boldly Together to New Frontiers of HIV Treatment

**DOI:** 10.9745/GHSP-D-14-00243

**Published:** 2015-03-02

**Authors:** Matthew Barnhart, James D Shelton

**Affiliations:** a Global Health: Science and Practice, Associate Editor.; b Global Health: Science and Practice, Editor-in-Chief.

## Abstract

New antiretrovirals (ARVs), particularly the potentially “game-changing” ARV dolutegravir, offer major potential to meet the compelling need for simpler and better HIV treatment for tens of millions of people in the coming decade. Advantages include substantially lower manufacturing cost, fewer side effects, and less risk of resistance. But key obstacles must be addressed in order to develop and introduce new ARVs in specific combinations optimized for the needs of low- and middle-income countries. Strong leadership will be essential from the global health community to nurture more focused collaboration between the private and public sectors.

## THE CRUCIAL NEED FOR BETTER ANTIRETROVIRAL THERAPY, ESPECIALLY FOR HIGH-BURDEN COUNTRIES

The current and projected need for HIV treatment is simply stunning. Already, among the estimated 35 million people living with HIV, 13.6 million are receiving antiretrovirals (ARVs),^1^ with about 3 of every 4 people on antiretroviral therapy (ART) residing in sub-Saharan Africa.^2^ However, achieving expanded 2013 treatment guidelines from the World Health Organization (WHO) would require ART provision to about twice the current number. And many have recommended that *all* people living with HIV infection should receive ARVs. Moreover, over time as survival improves substantially, the number of people living with HIV will increase markedly. Additional special demands are ARVs for people for whom first-line therapy has failed, people with tuberculosis (TB), pregnant and lactating women, and children.

At almost US$10 billion per year,^2^ international funding for HIV is the lion's share of all global health funding, and within that, support for HIV treatment is also the lion's share. That level of support seems to have plateaued, and indeed pressure is mounting for resource-constrained countries affected by HIV to take over more of the burden. Actually, more of the site-level costs of providing treatment comes from service delivery costs than from costs of the ARVs themselves; provision of services for established patients (excluding the cost of ARVs) averages $177 (range, $129–$235) per patient year (ppy)^3^ compared with $122 ppy^4^ for generic versions of the WHO-preferred first-line ARV combinations. Furthermore, patient compliance and continuation with current ARV regimens are far from optimal.

All this argues for ARVs that are not only cheaper but also more patient- and service delivery-friendly and efficient. Importantly, in addition to the benefits for combatting HIV, simpler and less expensive ART should ultimately reap tremendous rewards for global health more broadly, freeing up human and financial resources for overstretched health systems to address other pressing needs.

Less expensive and more practical ARVs are needed to meet the growing treatment needs in resource-constrained countries.

The ability to simplify ART delivery and lower cost is constrained by characteristics of the available ARV regimens, including their resistance barrier, safety, tolerability, efficacy, toxicities, monitoring requirements, and cost of manufacturing. One key constraint is that at least 3 ARVs must typically be provided to promote treatment effectiveness and help forestall the development of drug resistance. To maximize compliance, these are optimally combined in a single daily tablet. Currently, the WHO-preferred first-line ART regimen is efavirenz, tenofovir disoproxil fumarate (TDF), and either lamivudine or emtricitabine. While offering major advantages over previous regimens, it remains less than ideal due to its suboptimal barrier to development of resistance, potential for renal toxicity, neuropsychiatric side effects, possible interaction with contraceptive implants, and relatively high cost. In terms of second-line regimens, cost is a major barrier because they involve the much more expensive-to-manufacture protease inhibitors, with the generic regimens costing over $300 ppy.^4^ The numbers of patients requiring these regimens will increase dramatically over the next decade. Furthermore, with guidelines progressively recommending earlier initiation of ART, edging ever closer to a universal “test-and-treat” approach, the bar for regimen tolerability will rise further, since even relatively minor side effects may reduce willingness of patients who are clinically well to initiate, adhere to, or remain on lifelong treatment.

## DOLUTEGRAVIR: A POTENTIAL “GAME-CHANGER” FOR BETTER ARV REGIMENS

Thankfully, a next generation of better ARVs is on the horizon (Table 1), and some of these individual ARVs could be combined in regimens that should be easier to implement, less toxic, more durable, and also substantially less expensive to manufacture (Table 2). Leading the way for this new generation is dolutegravir, a recently approved ARV that belongs to a class of agents called integrase inhibitors, which block the last stage of the viral life cycle in which the viral genome integrates into the host DNA. However, dolutegravir differs from its prior integrase cousins, raltegravir and elvitegravir, and indeed from all other previously approved ARVs, in having a constellation of attributes that taken together could create a firm foundation for new “game-changing” combinations to facilitate the expanded roll out of simplified, less expensive ART.

**Table 1.** Three Promising ARVs of the Next Generation

**Drug Name**	**Innovator Company**	**Development Stage**	**Potential Advantages**
Dolutegravir	ViiV	Approved	Very high barrier to resistance and better regimen durability Reduced manufacturing cost Reduced side effects compared with efavirenz Potential for first- or second-line treatment More rapid decrease in viral load may increase efficacy for prevention of mother-to-child transmission among women initiating ART late in gestation Low dosage convenient for pediatric formulations May not reduce efficacy of progestin-containing contraceptive implants, which is a possible concern with efavirenz
TAF	Gilead	Late Phase III	Lower risk of renal and bone toxicity than TDF Lower manufacturing cost than TDF Potential for first- or second-line treatment, with possible greater efficacy than TDF for viruses resistant to some nucleosides/nucleotides
Doravirine	Merck	Early Phase III	Lower rates of central nervous system adverse events than efavirenz reported in phase II studies Possible low manufacturing cost given low dose May have better activity against many non-nucleoside reverse transcriptase resistant isolates common in sub-Saharan Africa, leading to possible utility in second-line combinations Characteristics (lack of food requirement, shorter half-life, lower potential for TB medication interactions) might make it a better partner for dolutegravir than rilpivirine as part of a simplified, low-cost 2-drug maintenance regimen

Abbreviations: ART, antiretroviral therapy; ARV, antiretroviral; TAF, tenofovir alafenamide fumarate; TB, tuberculosis; TDF, tenofovir disoproxil fumarate.

**Table 2.** Currently Available and Improved Hypothetical Combination ARVs

**ARV Drug Combinations**	**Innovator Companies**	**Estimated Cost per Year (US$)^a^**	**Comments**
**Current WHO-Recommended ARV Regimens for Adults**			
Efavirenz + TDF + lamivudine or emtricitabine (Atripla is a comparable pill marketed in the US)	NA (generic available)	$122	Preferred first-line regimen Highly effective and well tolerated Drawbacks include weak resistance barrier, potential for kidney and bone toxicity, neuropsychiatric side effects, possible interaction with contraceptive implants, and greater incidence of severe adverse birth outcomes in pregnancies receiving TDF-containing regimens compared with zidovudine-containing regimens
Lopinavir/ritonavir or atazanavir/ritonavir + zidovudine^b^ + lamivudine	NA (generic available)	$305–$318	Preferred second-line regimen Not available as a fixed-dose combination Resistance barrier suboptimal due to lack of second highly potent and robust agent to couple with the protease inhibitor Drug-drug interactions increased by ritonavir
**Highly Effective Alternatives, Not Ideal for Resource-Limited Settings**			
Elvitegravir + TDF + emtricitabine + cobicistat (Stribild, the “quad” pill)	Gilead	$184	Elvitegravir's resistance barrier is less robust than dolutegravir's Cobicistat increases drug-drug interactions and cost Bone and kidney toxicity possible with TDF A second-generation “quad” with TAF is in development and should decrease side effects and cost to a degree
Dolutegravir + abacavir + lamivudine (Triumeq, the “tri” pill)	ViiV	$179	Abacavir is expensive to manufacture and is associated with a rare but potentially life-threatening hypersensitivity syndrome Has utility for pediatrics due to abacavir not affecting bone
**Illustrative Improved (Hypothetical) 3-Drug Regimens**			
Dolutegravir + TAF + lamivudine or emtricitabine	ViiV + Gilead	$60	Higher resistance barrier and durability Reduced risk of renal and bone toxicity and manufacturing cost with TAF compared with TDF Primarily for first-line treatment, although it might have good activity as second-line for some patients
Dolutegravir + boosted protease inhibitor + TAF	ViiV + Gilead + Others	$266–$357 (or more^c^)	Improved second-line regimen, with a very robust resistance barrier that could make the need for third-line regimens very rare
Dolutegravir + TAF + doravirine	ViiV + Gilead + Merck	$64	Might be effective for first- or second-line treatment as doravirine has *in vitro* activity against non-nucleoside reverse transcriptase–resistant isolates common in sub-Saharan Africa
**Illustrative Improved (Hypothetical) 2-Drug Maintenance Combinations (for use after undetectable viral load achieved)**			
Dolutegravir + rilpivirine	ViiV + Janssen	<$40	Excellent tolerability with minimal side effects Both agents are already approved, allowing for expedited development Proof of concept for efficacy available from LATTE study Downsides include interactions with TB regimens, food requirement, and suboptimal efficacy for second-line treatment Currently in late stage clinical development that could lead to licensure
Cabotegravir + rilpivirine (long-acting injectable)	ViiV + Janssen	$40	Injection every 1–2 months may reduce risk of resistance due to non-adherence and increase confidentiality and quality of life If patients are lost to follow-up, risk of resistance could be much higher than an oral formulation Other advantages and disadvantages may be similar to dolutegravir/rilpivirine oral combination above Currently in phase II development
Dolutegravir + doravirine	ViiV + Merck	$50	Doravirine's different resistance profile, lack of food requirement, shorter half-life, and possibly lower potential for TB medication interactions might make it a better partner for dolutegravir than rilpivirine as part of 2-drug regimen Might have efficacy in first- and/or second-line treatment This is a hypothetical combination, not in development, and doravirine is now in early phase III development
Dolutegravir + lamivudine	ViiV	$46	Might be effective and well tolerated in first-line treatment, preserving many second-line options This is a hypothetical combination, not in development, but because one company makes both medicines, fewer obstacles might exist to develop it
Dolutegravir + TAF	ViiV + Gilead	$39	Hypothetical combination for first-line treatment, with some possible efficacy in second-line Currently not in development
Dolutegravir + boosted protease inhibitor	ViiV + Others	$252–$343 (or more^c^)	Hypothetical combination, not currently in development Could provide a very robust resistance barrier for second-line treatment

Abbreviations: ARV, antiretroviral; NA, not applicable; TAF, tenofovir alafenamide fumarate; TB, tuberculosis; TDF, tenofovir disoproxil fumarate; WHO, World Health Organization.

a For approved and available ARVs, these costs were based upon the generic prices from the Supply Chain Management Systems E-catalog, November 2014 version.^4^ Costs do not include in-country logistic and storage costs. While these prices are more than the manufacturing cost, they are used as a proxy for the fully loaded costs, given that the generic market is competitive with a relatively low profit margin. For hypothetical ARV combinations, illustrative manufacturing costs were estimated as follows: (1) rilpivirine oral or injectable: $15, based upon expert consultation,^6^ and assuming cost of oral rilpivirine ≤ cost of injectable; (2) dolutegravir oral: $25, based upon expert consultation,^6^ and assuming cost of oral dolutegravir ≤ cost of injectable; (3) cabotegravir injectable: $25, assumed to be same cost as dolutegravir injectable and given similar structure of the 2 compounds; (4) TAF: $14 assumes TAF costs 1/3 of TDF at approximately 1/10 the dose; (5) cobicistat: $90; in the absence of other data, cost assumed to be the same as another pharmacokinetic booster, ritonavir^25^; and (6) elvitegravir (150 mg/day) and doravirine (100 mg/day), both assumed to cost $25 per year to manufacture in the absence of other data. Note that the costs for these hypothetical ARVs are solely meant to provide a sense of the degree of savings that might be possible; actual costs may be substantially higher or lower than these estimates.

b WHO recommends that in cases in which zidovudine has been used in a first-line regimen, TDF should be included in the second-line regimen rather than zidovudine.

c Darunavir/ritonavir has the most robust resistance barrier of any boosted protease inhibitor but is not currently as widely available as a generic as lopinavir/ritonavir or atazanavir/ritonavir. Darunavir/ritonavir may be somewhat more expensive to manufacture.

To begin with, dolutegravir has a very high barrier to drug resistance,^5^ which is particularly advantageous in low-income countries where resistance testing is not widely available and treatment options are limited. Dolutegravir can also be manufactured at scale very inexpensively, given its low dose of only 50 mg once daily for treatment-naive patients.^6^ Additionally, dolutegravir is very well tolerated, with lower discontinuation rates due to side effects than efavirenz,^7^ the common component of WHO-preferred first-line regimens. Furthermore, dolutegravir might be included as a component of improved second-line ART regimens. Along with these salient features, dolutegravir also has other characteristics that may be important to address the special needs of women and children (Table 1).

But a key obstacle is the needed marriage with other ARVs in a fixed-dose tablet. Dolutegravir has not yet been approved, or even tested for that matter, in a fixed-dose combination (FDC) with 2 other ARVs such as TDF and lamivudine that have favorable characteristics for implementation in low-income countries. Its developer, ViiV Healthcare (an HIV-focused company jointly owned by GlaxoSmithKline, Pfizer, and Shionogi), has developed its own “tri” pill, Triumeq, which contains dolutegravir, abacavir, and lamivudine, 3 medications for which it controls the rights. However, abacavir has some downsides from the perspective of resource-limited settings, especially its very high manufacturing cost, with generic versions priced at $133 per year as a single agent.^4^ Therefore, for dolutegravir to realize its full potential for resource-constrained settings, it needs to find other partner ARVs beyond abacavir. Another downside to dolutegravir is that if given with the TB treatment drug rifampicin, it will require 50 mg twice-daily, rather than a once-daily dose.^8^

### Tenofovir Alafenamide: A Promising Partner for Dolutegravir

Tenofovir alafenamide fumarate (TAF) is a next-generation ARV that has been recommended by experts to be considered for improved combinations with dolutegravir.^9^ TAF's close relative, tenofovir disoproxil fumarate (TDF), is a common component of both first- and second-line treatment. But TAF is a much more potent prodrug than TDF: At a dose less than one-tenth of TDF, TAF produces intracellular levels of the active metabolite tenofovir diphosphate that are sevenfold higher.^10^ This enhanced potency offers a number of advantages, including reduced manufacturing cost, less risk of renal and bone toxicity than TDF,^11^ and also possibly greater activity in second-line regimens. In addition, with the recently announced results of the PROMISE study, which found that pregnancies in which women received a TDF-containing regimen were associated with significantly greater risks of severe adverse birth outcomes than pregnancies in which women received a zidovudine-containing regimen,^12^ the need to reduce toxicity of current TDF-containing regimens in resource-limited settings takes on greater importance, given the enormous numbers of pregnant women who receive ART in these countries.

Following the understandable proprietary route, Gilead, the developer of TAF, has been developing it in the context of a second-generation “quad” pill, combining TAF with 3 of its own compounds: the integrase inhibitor elvitegravir, emtricitabine, and a pharmacokinetic “booster,” cobicistat, which has no anti-HIV activity but is needed to increase elvitegravir levels. Although this “quad” is expected to be highly effective and well tolerated for first-line treatment, it has a few prominent disadvantages. First, compared with dolutegravir, elvitegravir has a less robust resistance barrier.^13^ Second, the pharmacokinetic booster cobicistat not only creates greater potential for drug-drug interactions but also substantially increases manufacturing costs.

### Obstacles to Combining Dolutegravir and TAF

Combining dolutegravir and TAF, along with a third agent (either lamivudine or emtricitabine), in a single FDC could be one way forward to a better first-line combination for developing countries. Unfortunately, these 2 drugs are owned by 2 competing pharmaceutical companies.

Combining dolutegravir with TAF (and a third agent) could be one way forward to a better first-line combination ARV.

Still, a mechanism exists to help with such situations. The Medicines Patent Pool (MPP) was created to enable originator (sometimes called innovator) companies to license out existing or new ARV agents to generic manufacturers.^14^ The intended advantage of the MPP was not only that new individual ARV agents could be manufactured at low cost more rapidly by generic producers, but also that having licenses from multiple companies in the pool might enable the creation of better, low-cost ARV combinations specifically tailored to the needs of developing countries.

The good news is that in 2014 the MPP finalized individual agreements for TAF and dolutegravir that in principle provide for the development of a combination product for developing countries.^15^^,^^16^ But celebrating such a marriage would be premature at this point, as a closer examination reveals some remaining steps that will be needed for these 2 ARVs to come together in a single pill (Table 3).

**Table 3.** Barriers to Developing a Combination ARV With Dolutegravir and TAF^a^

**Obstacle**	**Explanation**
Need for a company to manage the development and introduction of a new product with good collaboration from the other companies	If Gilead and/or ViiV are not willing to take leadership, generic companies or other partners might take the lead.
Access to data on each individual agent	Gaining regulatory permission to test agents together may require ability to reference the master file for each agent.
Financing of new product development	The cost of developing and introducing a new product would be substantial. Neither innovator nor generic manufacturers may have motivation to invest unless they have proprietary rights.
Formulation development	Combining the 2 agents into a single pill would require reformulation and expertise in this area.
Clinical testing of new combinations	Beyond showing bioequivalence of a combination to each agent given separately, further tests may be needed.
Manufacturing scale-up	A new, quality-assured process would need to be developed to manufacture the new product at large scale.
Registration of the products in many countries	A market authorization holder of the new product(s) would be needed, responsible for regulatory filing, marketing, and pharmacovigilance of the new product.
Introduction of the product in country programs	Country guidelines, policies, and plans would need to be adapted, and providers would need to be trained in how to use the new product.

Abbreviations: ARV, antiretroviral; MPP, Medicines Patent Pool; TAF, tenofovir alafenamide fumarate.

a Plus a third agent—either emtricitabine or lamivudine.

First, the details of such an arrangement would need to be ironed out, with good collaboration on all sides, including a plan for how this new combination would be manufactured and registered in many countries. Developing a novel formulation containing TAF and dolutegravir would require investment of additional time and resources, and the process of registering such a combination may in some cases require that the individual agents receive regulatory approval in specific countries first, which typically requires access to the master file for each agent. Accomplishing such tasks would likely be most efficient with active collaboration between Gilead and ViiV, although generic companies could take on these roles if given access to the necessary data and if they were confident enough in a return on investment to make the necessary upfront investments in formulation, manufacturing, and registration. Indeed, some precedent for generic firms taking the lead on such tasks comes from the early stages of HIV treatment roll out, in which these companies developed and gained rapid regulatory approval for novel FDCs that were not used in affluent countries, such as Triomune (stavudine, lamivudine, and nevirapine).

## REDUCING TO JUST 2 ARVS AT A TIME: A SIMPLER APPROACH

On a different front, ViiV is now collaborating with Janssen (owned by Johnson & Johnson) to develop a novel 2-drug FDC of dolutegravir (50 mg daily) and the non-nucleoside reverse transcriptase inhibitor (NNRTI) rilpivirine (25 mg daily) as a simplified maintenance therapy for patients who have already achieved undetectable viral load on a 3-drug regimen.^17^ While some other 2-drug ART regimens that have previously been tested as *initial* treatment of HIV have proven inferior to 3-drug ART,^18^ strong proof of concept for this 2-drug *maintenance* approach was provided by data from the Long-Acting Antiretroviral Treatment Enabling (LATTE) study. The LATTE study was a phase IIb trial that reported high efficacy of a 2-drug oral maintenance therapy with rilpivirine and another integrase inhibitor cabotegravir (formerly known as GSK744),^19^ which is closely related to dolutegravir. Although the LATTE study was designed to lay the groundwork for a once-monthly long-acting injectable combination of rilpivirine and cabotegravir, the plans to develop dolutegravir and rilpivirine together in a combination tablet make it much more likely that a 2-drug oral maintenance combination might be available relatively soon, since both of these ARVs are already approved as individual agents.

Why might such a “bi” be beneficial as maintenance therapy compared with the current standard of triple-drug therapy? First, both dolutegravir and rilpivirine have excellent tolerability, without the bone or renal toxicities of TDF or the neuropsychiatric side effects of efavirenz. Second, the cost to manufacture this low-dose, 2-drug combination would be extremely low, probably less than $40 per year,^6^* or less than one-third the cost of current first-line regimens, a key point that seems to have gone largely unnoticed so far. Another potential advantage of using only these 2 agents is that it spares nucleosides/tides in first-line regimens, which could possibly create more options for second- and third-line treatment. However, on the downside, rilpivirine cannot be administered with the TB medication rifampicin and also must be taken with food. Given dolutegravir's very strong resistance barrier and potency, there may be other possible partners for dolutegravir as part of 2-drug maintenance first-line combinations that are worthy of exploration, including, for example:

- Doravirine (Merck), an NNRTI effective at a low dose of 25–100 mg daily that is now entering phase III development^20^ (Table 1)
- TAF (if obstacles to collaboration could be overcome)
- Even possibly lamivudine, an approved nucleoside that is already included with dolutegravir as part of the 3-drug regimen Triumeq

## IMPROVED SECOND-LINE COMBINATIONS WITH DOLUTEGRAVIR

In addition to strong potential for improving first-line treatment, dolutegravir also creates many new possibilities for better second-line treatment regimens. The potential advantages of dolutegravir as a component of either first- and/or second-line regimens raise the question of whether it should be prioritized for patients who are treatment-naive, treatment-experienced, or both. It would be a mistake at this juncture to assume in the absence of clear data that dolutegravir should be reserved for only one or the other. Rather, studies should be designed to answer specific questions regarding how best to maximize the value of dolutegravir via optimal sequencing of the various possible first- and second-line regimens.

Dolutegravir also holds promise for better second-line ARV combinations, in addition to improving first-line treatment.

Just as “bi” combinations of dolutegravir with an NNRTI would seem to hold promise for maintenance first-line therapy, 2-drug combinations of dolutegravir with boosted protease inhibitors could be envisioned for second-line maintenance therapy. In light of the very high resistance barriers of both of these classes of agents, such combinations would be expected to be extremely durable, making the likelihood of eventually requiring a third-line regimen very low. However, as the cost would still be quite substantial due to the protease inhibitor component, it should also be a priority to explore other non-protease inhibitor second-line regimens that might be possible by combining dolutegravir with other new agents as these become available. For example, doravirine has good in vitro activity against viruses that have developed resistance to other NNRTIs used in first-line treatment, such as efavirenz or rilpivirine.^21^ Therefore, doravirine might merit exploration as a component of a 3-agent second-line regimen with dolutegravir and other existing ARVs that may have activity in this context, such as TAF or zidovudine.

## LONG-ACTING INJECTABLE ARVS

Another exciting frontier for future generations of ARVs is long-acting injectable agents, including the 2 aforementioned nanosuspension formulations of cabotegravir and rilpivirine, which are being developed as a maintenance therapy to be given every 1, or possibly 2, months. The promise of injectable ART is that it could ensure adherence, which might dramatically reduce the risk of resistance among patients who are retained in follow-up, thereby also possibly reducing the need for lab monitoring for virologic failure.

ART in an injectable form could improve adherence and reduce the risk of drug resistance.

On the other hand, the long-acting nature of these formulations could be a double-edged sword; patients who are lost to follow-up (which happens at high rates over time in most ART programs) would experience a long tail of suboptimal drug levels lasting for months, rather than days with typical oral formulations, which might increase, rather than decrease, the overall risk of resistance. Cost of goods could also be a relative downside, because although the manufacturing costs of these injectable formulations may be reasonable at scale, they still might be expected to be somewhat more expensive to manufacture than oral formulations of the same medications. The high injection volumes required for the current agents is another potential drawback, which may decrease tolerability and willingness of some patients to opt for an injection.

Nevertheless, over the longer-term, injectable ART regimens are undoubtedly an area with great potential to simplify and improve ART. Further investments in discovery and earlier stage research are warranted to enable more rational design of optimal agents and delivery systems that would offer advances compared with the current agents in clinical development. In the medium-term, the largest impact of injectable ARVs in resource-limited settings may be for preexposure prophylaxis (PrEP). The cabotegravir nanosuspension injection has been shown to be completely protective against acquisition of simian/human immunodeficiency virus (SHIV) in low-dose viral challenge monkey models,^22^^,^^23^ is cold-chain independent, and has pharmacology in humans compatible with dosing once every 3 months.^24^ With HIV incidence rates remaining shockingly high in some populations, including young women under age 25 in several countries in eastern and southern Africa, who also have relatively high use of injectable contraceptives (typically also given every 3 months), such an agent could potentially have a very large public health impact if proven safe and effective.

## JOINT ACTION NEEDED TOWARD BETTER ARV REGIMENS

The HIV community can take great pride in its remarkable history of collaboration for the common goal to combat AIDS. Actors such as activists, researchers, United Nations agencies, the Global Fund to Fight AIDS, Tuberculosis and Malaria, government funding and regulatory agencies, foundations, governments of highly-affected countries, pharmaceutical companies, UNITAID, the Clinton Health Access Initiative, and the MPP have successfully taken action together to enable access to better ARV regimens and to build consensus on what new ARVs should be highest priority.

Now is the time to push the envelope even further. It is also time for other voices from the broader global health community to enlist their support, recognizing that development of better ARV regimens can have profound implications not only for HIV objectives but also for the feasibility of other health programs to ultimately reach their goals. As the above examples hopefully illustrate, the promise of the next generation of ARVs is bright. However, more focused collaboration and concrete steps are needed for this technical potential to be fully realized in the rapid timeframe that is required. And the free market penchant toward proprietary products must be balanced by the compelling need to come together to make this next generation of better ARVs available soon where they are needed most.

Development of better ARV combinations can help meet HIV as well as other health program objectives.

Strong leadership will be essential to take on this challenge and to nurture a more substantive, goal-driven level of collaboration between the public, private, and academic sectors. Active participation of originator pharmaceutical companies will be critically important, as these companies have the strongest capacity for developing new products. Pharmaceutical companies have taken a big step forward by licensing compounds to the MPP, but now is the time for these companies to step up to the plate and exercise further leadership in precisely those areas that are in their “wheelhouse”—research and product introduction—demonstrating on a grand scale the importance of their innovative capacity for global heath. At the same time, the public sector can complement such efforts, providing every reasonable incentive for private-sector companies to collaborate and supporting activities where gaps remain. Given that substantial barriers exist for the private sector in cases where potential for a strong profit may not be evident (Table 3, for example), additional public investment would likely be needed to help expedite the development of ARV regimens optimized for developing countries.

Public investment would likely be needed to help expedite the development of combination ARVs optimized for developing countries.

Building on prior efforts such as the Conference on Antiretroviral Dose Optimization II,^9^ public and private partners should come together to agree on a *short* list of “best bets” for better ARV regimens for developing countries, including combinations involving dolutegravir, TAF, doravirine, and possibly other new ARVs. A concrete development pathway should then be formulated with clear go/no-go criteria and timelines for expedited testing of these “best bets,” including realistic plans and mechanisms to address any obstacles to combining specific promising agents. Research is needed not only to enable regulatory approval of better products but also to inform policy on how to use such new ARV combinations optimally, including especially how to sequence various possible regimens as first- and second-line treatments, and giving adequate attention to the needs of special populations such as pregnant women and children. Gathering more data on long-term safety of the new agents among populations in developing countries is also important. In addition to these investments in research, well-coordinated support is needed to address bottlenecks across the value chain to introduction of new ARV products, including registration, financing, marketing, manufacturing, and global and country-level policy and planning issues. As dolutegravir is already approved and seems to have the most “game-changing” potential of any of the next-generation ARVs, a high priority should be put in the near-term on researching how to optimally use this agent and on expediting its registration and introduction in countries.

Effectively accomplishing the complex and interconnected set of research, development, and introduction efforts envisioned above would require a *strong coordination function*, through an organization or team focused on bringing the willing partners together. Estimating the cost of the activities required, accounting for what resources are already available, and identifying how funding could be provided and by whom to fill remaining gaps would be essential to better inform the diverse set of partners who may be able to contribute to these efforts. Although the cost of needed activities would undoubtedly be substantial, the potential return on investment would be very high. Furthermore, the absolute amount of additional funds needed would be relatively small compared with what is currently being spent on HIV treatment, with the basic costs of developing the individual agents already being covered by the originator companies.

## CONCLUSION

We as a global health community have an opportunity to take concrete steps to accelerate development and introduction of better drug combinations critical to ART access and scale-up. If we focus and act collaboratively, we can confidently expect that better ARV regimens will arrive sooner, rather than later. The benefits would be enormous, not only for the tens of millions of people who will need ART in the coming decades but also for countless others at risk from non-HIV-related conditions, as increasing efficiencies in HIV service delivery create opportunities for greater scale-up of the wide range of public health interventions needed by all.

Note: Meaningful changes were made to this article since publication of the “Advance Access” version.

## Footnotes

* This cost estimate is derived from a consultation including manufacturing experts, which estimated a cost per year of $25 and $15 for hypothetical injectable formulations of rilpivirine and dolutegravir, respectively. The overall cost of an oral formulation is assumed to be in a similar range or less, although admittedly the dose of active pharmaceutical ingredient required for an injectable formulation may be lower than for an oral formulation.

## Notes

**First published online:** 2015 Jan 28
